# GLP-1 RAs and SGLT2i: two antidiabetic agents associated with immune and inflammation modulatory properties through the common AMPK pathway

**DOI:** 10.3389/fimmu.2023.1163288

**Published:** 2023-11-20

**Authors:** Alessio Mazzieri, Giuseppe Basta, Riccardo Calafiore, Giovanni Luca

**Affiliations:** ^1^ Translational Medicine and Surgery, Department of Medicine and Surgery, University of Perugia, Perugia, Italy; ^2^ Division of Internal Medicine and Endocrine and Metabolic Sciences (MISEM), Laboratory for Endocrine Cell Transplants and Biohybrid Organs, Department of Medicine and Surgery, University of Perugia, Perugia, Italy; ^3^ Diabetes Research Foundation, Confindustria Umbria, Perugia, Italy; ^4^ Department of Medicine and Surgery, University of Perugia, Perugia, Italy; ^5^ Division of Medical Andrology and Endocrinology of Reproduction, Saint Mary Hospital, Terni, Italy

**Keywords:** Tregs, Foxo, AMPK pathway, SGLT2I, GLP1-RAs, inflammation, type 2 diabetes

## Abstract

Immune cells and other cells respond to nutrient deprivation by the classic catabolic pathway of AMPK (Adenosine monophosphate kinase). This kinase is a pivotal regulator of glucose and fatty acids metabolism, although current evidence highlights its role in immune regulation. Indeed AMPK, through activation of Foxo1 (Forkhead box O1) and Foxo3 (Forkhead box O3), can regulate FOXP3, the key gene for differentiation and homeostasis of Tregs (T regulators lymphocytes). The relevance of Tregs in the onset of T1D (Type 1 diabetes) is well-known, while their role in the pathogenesis of T2D (Type 2 diabetes) is not fully understood yet. However, several studies seem to indicate that Tregs may oppose the progression of diabetic complications by mitigating insulin resistance, atherosclerosis, and damage to target organs (as in kidney disease). Hence, AMPK and AMPK-activating agents may play a role in the regulation of the immune system. The connection between metformin and AMPK is historically known; however, this link and the possible related immune effects are less studied about SGLT2i (Sodium-glucose co-transport 2 inhibitors) and GLP1-RAs (Glucagon-like peptide-1 receptor agonists). Actual evidence shows that the negative caloric balance, induced by SGLT2i, can activate AMPK. Conversely and surprisingly, an anabolizing agent like GLP-1RAs can also upregulate this kinase through cAMP (Cyclic adenosine monophosphate) accumulation. Therefore, both these drugs can likely lead to the activation of the AMPK pathway and consequential proliferation of Tregs. These observations seem to confirm not only the metabolic but also the immunoregulatory effects of these new antidiabetic agents.

## 1 Introduction

In the context of T regulatory lymphocytes, innate Treg (nTreg, or natural Treg, which leaves the thymus in the form of mature cells) and acquired Treg (iTreg, induced Treg, generated by naive CD4+ T response to antigenic stimulation) have been characterized (1). These cell types may exert their immunoregulatory activity directly by cell-to-cell contact, releasing immunosuppressive cytokines, or induction of apoptosis (1). Specifically, the suppression mechanisms of Tregs consist of:

1) Modulation of APC (Antigen-presenting cell). The interaction between CTLA-4 (Cytotoxic T-Lymphocyte Antigen 4) on Tregs and its ligand CD80/86 on APCs provides a negative signal for T cell activation (2).
2) Production of immunoregulatory molecules. The ecto-enzymes CD39 and CD73, expressed on Tregs, catalyze the metabolism of ATP (Adenosine triphosphate) into AMP (Adenosine monophosphate) and in turn, produce the immunoregulatory purine, adenosine (3). Furthermore, the anti-inflammatory cytokines produced by Tregs, such as IL-10 (Interleukin-10), IL-35 (Interluekin-35), and TGF-β (Transforming growth factor-beta), have been linked to the inhibition of T cell activation *in vivo* (1).
3) Induction of apoptosis. Tregs transfer cAMP to effector T cells with subsequent inhibition of NFAT (Nuclear factor of activated T-cells) and IL-2 (Interleukin-2) transcription and with subsequent cellular apoptosis (3). Moreover, Tregs may directly induce apoptosis via perforin, granzyme A/B, and the FasL/Fas pathway (4).

The pivotal action of Tregs in autoimmune diseases, such as T1D (Type 1 diabetes), is well established; whereas, their role in T2D (Type 2 diabetes), historically considered to not be an immune-mediated condition, has not been clarified yet (5). However, a persistent and dysregulated inflammatory state contributes to the development of systemic complications in T2D patients, such as atherosclerosis (6) and recent studies suggest that Tregs activity can control the progression of this vascular disorder (5). Moreover, Tregs have been found in both VAT (Visceral adipose tissue) and SAT (Subcutaneous adipose tissue). Their presence could be necessary for both suppression of adipose tissue-related inflammation, and helping maintenance of its homeostasis with clear benefits in terms of contrast to insulin resistance (5). Further evidence seems to suggest a role for Tregs in improving immunopathologic damage of target organs in diabetic complications: for example, a study of diabetic nephropathy in db/db mice (Leptin-Receptor deficient mice) showed that Tregs could attenuate T2D-related kidney morphologic and functional lesions (5). Possibly, Tregs may help suppress inflammation at multiple levels, with regard to T2D pathogenic pathways (5, 7).

FOXP3 (Forkhead box P3) is implicated in the role and the function of Tregs. This gene has provided relevant information with respect to the generation and maintenance of Tregs. Upon its expression, a self-regulating transcriptional loop stabilizes the expression of FOXP3 to strengthen Tregs differentiation and activate their suppressive function. However, these molecular mechanisms need to be further elucidated (8). Treg cell fate may be influenced by FOXP3 interaction with intermediates for activation of TCR (T cell receptor), such as IL-2 and TGF-β signaling pathways (9). Among the various regulatory forms of FOXP3, the Foxo (Forkhead box O) transcription factors (Foxo1 and Foxo3) play a key role (10). Ouyang et al. also reported that mice with T cell-specific deletion of both Foxo1 and Foxo3 developed fatal systemic inflammatory disease, due in part to functional defects in Foxp3+ Treg cells. They showed that Foxo1 and Foxo3 directly bind to the FOXP3 promoter region and transactivate its promoter activity, in a Foxo1-binding sequence-specific manner. All these reports indicate that Foxo family transcription factors are required for appropriate control of the expression of FOXP3. Indeed, the impairment in this Foxo-dependent gene expression in Tregs hampers their function, which may result in autoimmunity and systemic inflammation (11). Foxo transcriptional factors can act as either transcriptional activators or repressors by forming different molecular complexes with various transcriptional modulators including b-catenin, STAT3 (Signal transducer and activator of transcription 3), Runx3 (Runt-related transcription factor 3), and Smad3 (Mothers against decapentaplegic homolog 3). Additionally, their function is tightly regulated by the upstream PI3K-Akt (Phosphoinositide 3-kinase-Protein kinase B) pathway, which phosphorylates Foxo molecules and facilitates their nuclear export into the cytoplasm. In immune cells, the PI3K/Akt pathway is activated by several stimuli via specific receptors, including the BCR (B cell receptor), TCR, and cytokine and chemokine receptors. Upon antigen or cytokine stimulation, Foxo transcriptional factors are rapidly phosphorylated and deactivated in a PI3K-dependent manner, whereas cytokine withdrawal elicits their de-phosphorylation, activation, and consequential expression of FOXP3 (10). Moreover, the presence of AMPK (Adenosine monophosphate kinase) also seems to activate the Foxo transcriptional factors by direct phosphorylation. Indeed, this kinase, well-known for its metabolic pathways, could play a role in the control of inflammation; consequently, the AMPK-activating antidiabetic agents may likely affect the regulation of the immune system (12).

## 2 The AMPK pathway

AMPK is a serine/threonine kinase comprised of a catalytic alpha subunit and two subunits of beta and gamma regulators (13). This enzyme regulates the intracellular AMP/ATP ratio. If this ratio is too high, the amino acid threonine 172 available in its alpha chain is functionalized by the phosphorylated liver kinase B1 (LKB1), leading to AMPK activation (14, 15). At the cellular level as well as whole-body energy homeostasis, AMPK acts as an intracellular energy sensor and influences the regulation of glucose and fatty acid metabolism (13, 16, 17). Concentrations of AMPK usually rise when the consumption of energy exceeds its production. When glucose or ATP levels decline, AMPK is activated to phosphorylate various molecules. Particularly, this kinase can activate the Foxo transcription factors by phosphorylation (10). In mammals, the Foxo subfamily is comprised of four members: Foxo1, Foxo3, Foxo4, and Foxo6. They are important regulators of cell cycle progression, apoptosis, glucose metabolism, and stress resistance via integrating information associated with the presence of nutrients, growth factors, and other signals. Foxo1 and Foxo3, mainly expressed in immune cells, are essential for the transcription of FOXP3, and hence the homeostasis of Tregs (10). Under this perspective, AMPK plays a role in the immune system, although the link between AMPK and Tregs needs more clarification.

## 3 The relationship between AMPK and antidiabetic drugs

Metformin, the most widely used oral agent for the treatment of T2D, is historically known as an AMPK activator (18). Through this pathway, metformin can suppress gluconeogenesis and reduce blood sugar levels (19). Moreover, the AMPK pathway might be one of the related mechanisms to the known Tregs proliferation (20) induced by metformin. This immunoregulatory effect could be found also in the new antidiabetic agents but presently, their connection with AMPK is less known as compared to metformin.

### 3.1 SGLT2i and AMPK

**Figure 1:**
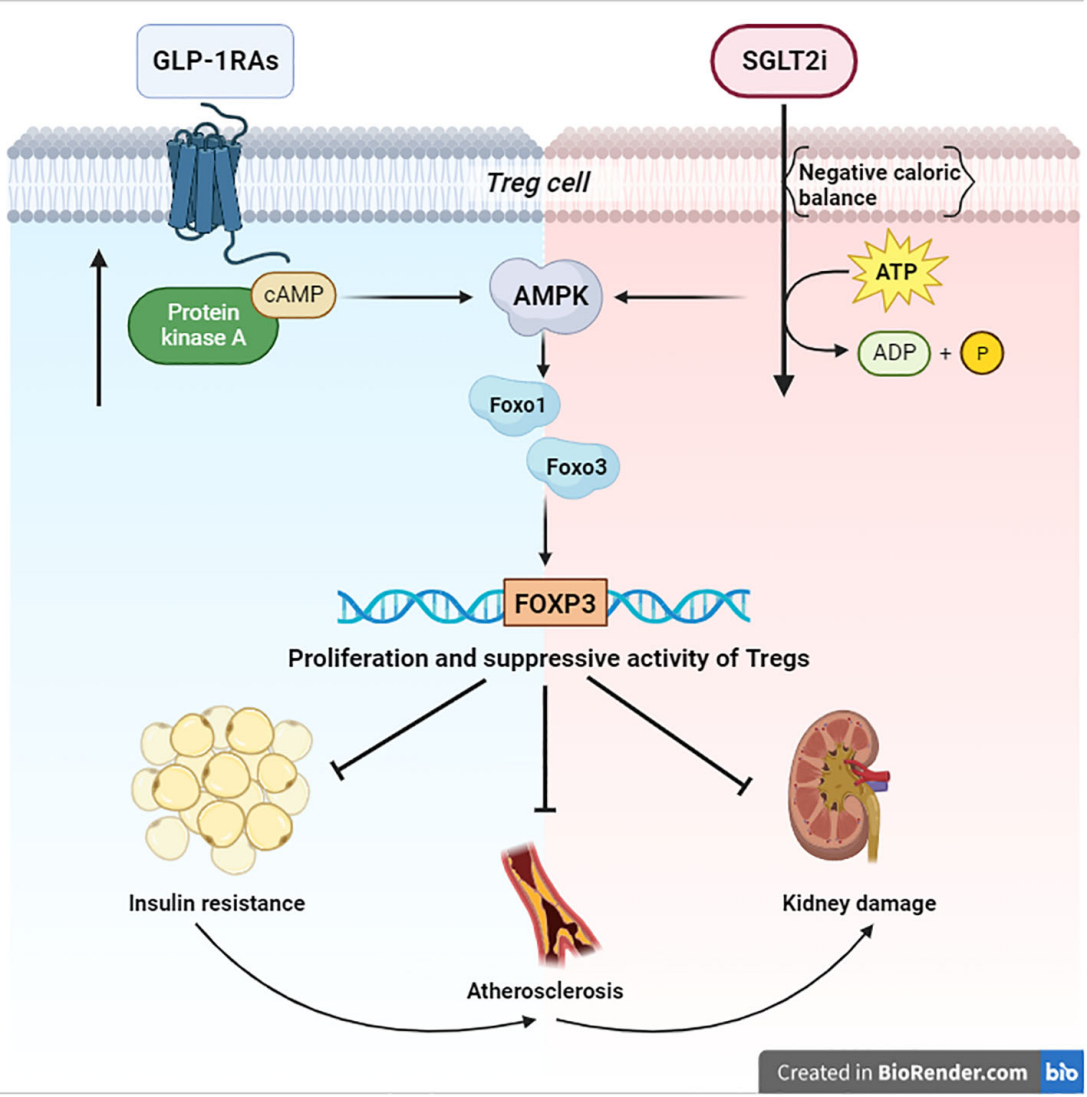
Signaling of AMPK/FOXO/FOXP3 Both anabolic (blue zone) and catabolic (red zone) signals converge on AMPK. This kinase activates the Foxo transcriptional factors by direct phosphorylation. Specifically, Foxo1 and Foxo3 are essential for the transcription of FOXP3 and the consequential proliferation of Tregs, which are necessary to suppress the damage of chronic inflammation in T2D. BioRender.com.

The SGLT2i (Sodium-glucose co-transport 2 inhibitors) are a class of antidiabetic agents, targeting the major glucose transporter SGLT2 in the kidney. SGLT2 inhibition reduces glucose reabsorption at the level of the proximal renal tubule, promotes urinary glucose excretion independent of insulin action, and causes a negative caloric balance (21, 22). Due to the induction of a nutrient deprivation state, SGLT2 inhibitors upregulate the energy deprivation sensor AMPK (23) and its immunoregulatory effects (**Figure 1**). A 6-month Empagliflozin treatment increased FOXP3 expression (compared to baseline) and then improved the protective function of Tregs; this effect may counteract the cardiovascular complications by the reduction of the systemic inflammation (24). In this regard, empagliflozin treatment has also been capable of reducing IL-1β (Interleukin-1 beta) secretion (25). Another study with canagliflozin showed the reduction of IL-1β, IL-6 (Interleukin-6), and MCP-1 (Monocyte chemoattractant protein-1); also in this case, these AMPK-mediated effects led to cardiovascular benefits through a reduction of chronic inflammation (26, 27). Moreover, dapagliflozin attenuated myocardial inflammation, fibrosis, apoptosis, and diabetic remodeling, likely by virtue of AMPK activation and its immune regulation effects (28, 29). However, current knowledge on immune modulation and proliferation of Tregs through AMPK by SGLT2i requires further study.

### 3.2 GLP1-RAs and AMPK

GLP-1 (Glucagon-like peptide 1) is an incretin hormone, secreted from L-cells of the small intestine in a glucose-dependent manner, that enables stimulation of insulin secretion, increases pancreatic β-cell mass, and inhibits glucagon secretion and gastric emptying, thereby reducing postprandial glycemia. Due to rapid degradation by DPP-4 (Dipeptidyl peptidase 4), the half-life of endogenous GLP-1 is very short; therefore, longer half-life synthetic analogs have been developed for clinical use, as a new class of antidiabetic agents (30).

The anti-inflammatory potential of GLP1-RAs (Glucagon-like peptide-1 receptor agonists) could depend on their direct effect on immune cells, an indirect effect caused by weight loss, or a combination of the two. A direct effect of GLP1-RA on immune cells would obviously require that they express the GLP1-R (Glucagon-like peptide-1 receptor) (31).

Several reports have suggested that GLP1-R signaling may regulate T cell subsets. Hadjyanni et al. demonstrated that GLP1-R activation leads to a modest but statistically significant increase in cAMP concentration in mixed leucocyte populations, including Tregs (32). Because increasing evidence suggests a link between cAMP and AMPK pathways (33), it is possible to speculate, following the activation of GLP1-R, on the onset of a cAMP/AMPK-dependent signaling (**Figure 1**). The relationship between AMPK and GLP1-RA is well-known with special regard to cardiovascular benefits (34). For instance, liraglutide increased AMPK phosphorylation in the heart of diabetic mice (35), inducing effects similar to those of metformin (36, 37). In another study, a short-term treatment with a weight-neutral dose of liraglutide could reverse the molecular pathophysiology of obesity-induced heart disease in mice through various mechanisms, among which a pivotal role is played by AMPK (38). Within the same context of cardiovascular complications, Guo’s study also showed that exenatide treatment increased the cAMP accumulation and the level of phosphorylation of AMPK in the hearts of diabetic rats (39). All GLP-1RAs significantly reduce myocardial tissue T cell accumulation, an effect that resembles one observed in the kidney; indeed, GLP-1 and its cleavage products have been shown to be renoprotective in murine diabetic nephropathy through the reduction of renal infiltration of inflammatory cells (40).

Moreover, the link between GLP1-R and cAMP generation has also been investigated with regard to the preservation of function in diabetic kidney disease. In the proximal tubule, the activation of GLP1-R leads to the onset of the cAMP signaling cascade with phosphorylation of NHE3 (Na^+^/H^+^ exchanger isotope 3) and reduction of its function; hence GLP1-R signaling can contribute to the regulation of sodium balance and maintain blood pressure in the normal range (41).

Further experiments are still required to elucidate the potential mechanisms by which GLP1-R activation increases Treg functions (31). Certainly, GLP1-R activation leads to cAMP accumulation, and GLP1-R signaling contributes to the proliferation of both thymocytes and peripheral Tregs (32).

However, the effects of GLP1-RAs on immune regulation still remain uncertain. Indeed, these drugs, as other anabolic factors, can activate PI3/Akt, which leads Tregs into Foxo and FOXP3 suppression (10). This evidence may appear unexpected; nonetheless, due to the proliferation of Tregs during treatment with GLP1-RAs, the activation of AMPK seems to be the prevailing pathway. With regard to pending doubts, some authors are in support of a possible downstream regulation of AMPK on PI3K/Akt (42) or alternatively believe that GLP1-RAs may exert negative effects on PI3K/Akt (43). Certainly, to investigate these open issues, further studies are warranted.

## 4 Discussion and conclusions

Cells respond to nutrient fluctuations by adjusting anabolic versus catabolic processes. When glucose or ATP levels decline, the AMPK becomes activated and promotes catabolic processes to restore energy homeostasis and attenuate cellular senescence (44, 45). Moreover, AMPK can activate the Foxo transcription factors, important regulators of cell cycle progression and resistance to stress by phosphorylation (12). In particular, the regulation of Foxo1 and Foxo3, expressed in immune cells, is essential for transcription of FOXP3, thereby for Treg homeostasis (10). Tregs are necessary to suppress the chronic inflammation in T2D and to fight insulin resistance, atherosclerosis, and damage to target organs, as in kidney disease (5, 7) (**Figure 1**). For this purpose, Tregs expansion could change the fate of diabetic patients. Consequently, the immunological impact of the new antidiabetic drugs (SGLT2i and GLP1-RAs) looks very relevant. Nevertheless, the link between their immunoregulatory effects and AMPK still remains obscure. The aforementioned evidence shows that a negative caloric balance, as induced by SGLT2i, activates AMPK (23). Modulation of these pathways by the SGLT2 inhibitors has been shown to alleviate metabolic diseases, attenuate vascular inflammation and arterial stiffness, improve mitochondrial function, and reduce oxidative stress-induced tissue damage (23) (**Figure 1**). However, an anabolizing agent such as GLP1-RAs can also upregulate the AMPK-pathway, due to cAMP accumulation (33) by GLP-1R stimulation. These findings help our understanding of the role of the GLP-1R in the Treg pool development/expansion, maintenance, and function (32) (**Figure 1**). Finally, both anabolic and catabolic signals seem to converge on AMPK and its immunoregulatory effects on Tregs. These cells are made to protect, and they are capable of carrying out their work under both anabolic and catabolic signaling. This may appear to be paradoxical; however, catabolic processes, after all, result in starving cells to spare nutrients, hence sustaining cellular processes, and maintaining survival. Probably, in light of the postulated role of AMPK in promoting cell survival, and its emerging role in re-establishing metabolic homeostasis of Tregs, it is conceivable that AMPK may be directly modulated by apparently contrasting signals. Due to the fact that AMPK promotes glucose and lipid homeostasis, the current findings suggest that AMPK-activating agents (metformin, SGLT2i, GLP1-RAs) may certainly help patients with type 2 diabetes. However, these benefits must be extended to the AMPK-related immunological effects. In this sense, the chance to employ a combination therapy could envision a new avenue with respect to the immunoregulatory effects of these agents. A major study of the precise mechanisms of action of these drugs could better explain their regulatory effect on chronic inflammation in type 2 diabetes.

## Author contributions

AM wrote the manuscript. GB contributed to the redaction of the review. RC and GL contributed as senior authors to the supervision of the final manuscript. All authors contributed to the article and approved the submitted version.
